# The Genetic Diversity and Structure of Linkage Disequilibrium of the MTHFR Gene in Populations of Northern Eurasia 

**Published:** 2012

**Authors:** E.A. Trifonova, E.R. Eremina, F.D. Urnov, V.A. Stepanov

**Affiliations:** Research Institute of Medical Genetics, Siberian Branch, Russian Academy of Medical Sciences; Buryat State University; University of California, Berkley, USA

**Keywords:** genome, linkage disequilibrium, populations of Northern Eurasia, methylenetetrahydrofolate reductase, haplotype

## Abstract

The structure of the haplotypes and linkage disequilibrium (LD) of the methylenetetrahydrofolate reductase gene (*MTHFR*) in 9 population groups from Northern Eurasia and populations of the international HapMap project was investigated in the present study. The data suggest that the architecture of LD in the human genome is largely determined by the evolutionary history of populations; however, the results of phylogenetic and haplotype analyses seems to suggest that in fact there may be a common “old” mechanism for the formation of certain patterns of LD. Variability in the structure of LD and the level of diversity of* MTHFR*haplotypes cause a certain set of tagSNPs with an established prognostic significance for each population. In our opinion, the results obtained in the present study are of considerable interest for understanding multiple genetic phenomena: namely, the association of interpopulation differences in the patterns of LD with structures possessing a genetic susceptibility to complex diseases, and the functional significance of the pleiotropic*MTHFR *gene effect. Summarizing the results of this study, a conclusion can be made that the genetic variability analysis with emphasis on the structure of LD in human populations is a powerful tool that can make a significant contribution to such areas of biomedical science as human evolutionary biology, functional genomics, genetics of complex diseases, and pharmacogenomics.

## INTroduction

Genetic variability underlies the human phenotypic variation and plays a significant role in explaining the differences between individuals in their susceptibility to complex diseases (CD) and in determining the metabolic pathways involved in the development of pathological processes. Single nucleotide polymorphisms (SNPs) represent the most common type of genome variability. Thanks to the efforts of the International SNP Consortium, ~ 10 million SNPs with an approximate density of 1 polymorphism per 300 bp have currently been identified [1]. Each new allele of a polymorphic variant emerges from an already existing haplotype, the ancestral variant of a particular marker originally being associated with its alleles. New haplotypes are formed via the accumulation of new mutations and recombinations. The coinheritance of alleles in a haplotype manifests itself at the population level as the linkage disequilibrium (LD).

At the time of writing, the architecture of LD in the human genome is the subject of active discussions and research [2–7]. It has been shown in a number of studies that blocks of associated sites demonstrating no signs of substantial recombination in the evolutionary history of our species can be distinguished in the genome. These blocks are delimited by regions with a higher rate of recombination, the so-called “hotspots” [8, 9]. The patterns of linkage disequilibrium in modern human populations are the result of complex evolutionary processes, including the demographic population history (alteration of the effective population size, structure of population stratification, and migrations), as well as gene-specific factors, such as the mutation and recombination rates and selection pressure. Analysis of the structure of LD enables to reconstruct the demographic history of modern populations and plays a key role in the mapping of the CD genes [10].

**Table 1:** Linguistic and anthropological characteristics of the populations

Ethnic group	Population, community	N	Locality	Linguistic classification, family/group	Race and anthropological type
Tuvinians	Settlement of Bai-Taiga	134	Tuva Republic	Altaic/Turkic	Mongoloid (Central Asian)
Buryats	City of Ulan-Ude,Settlement of Khuromsha	6060	Buryat Republic	Altaic/Mongolic	Mongoloid (Central Asian)
Yakuts	Settlement of Dyupsya	81	Sakha Republic	Altaic/Turkic	Mongoloid (Central Asian)
Kyrgyz	North (Settlements of Kegety, Taldy-Su),South (city of Osh)	85 111	Republic of Kyrgyzstan	Altaic/Turkic	Mongoloid (South Siberian)
Kets	Settlement of Kellog	38	Turukhanskii district,Krasnoyarsk krai	Paleo-Asian/Ket	Mongoloid (North Asian)
Khanty	Settlement of Russinskii	142	Surgut district,Khanty-Mansi Autonomous Okrug	Uralic/Finno-Ugric	Uralic (transitory)
Russians	City of Tomsk	126	Tomsk oblast	Indo-European/Slavic	Caucasian(East Caucasian)

Along with the whole-genome patterns of LD that have been investigated in modern genome-wide studies [11–15], the structure of LD in separate, functionally significant genomic sites (in particular, in the genetic loci associated with common human diseases) is of considerable interest. The importance of the analysis of the haplotype structure of these genomic regions is rooted in the necessity of revealing the functionally significant variants of these genes which make possible their participation in their common component of the inherited susceptibility to CD, on the one hand, and to the significance of the assessment of the evolutionary genetic mechanisms of the generation of genetic variability in these genomic loci, on the other hand. These mechanisms were presumably formed with allowance for such factors as natural selection, genetic drift and migration, as well as via comparison of the genetic-demographic scenarios obtained by the analysis of the fine structure of the candidate genes of CD with those based on the data of conventionally neutral genetic systems and the results of whole-genome studies.

**Table 2:** Characteristics of the *MTHFR* gene SNPs studied

№	SNP	Position on a chromosome (according to the NCBI database)	dbSNP alleles	Ancestral allele	Mutation type	Localization in the*MTHFR*gene(according to data from the NCBI database)
1	rs3753588	11863904	A/G	G		Intron 1
2	rs2066470	11863057	C/T	C	Synonymous (39 Pro/Pro)	Exon 2
3	rs17037397	11862163	А/С	C		Intron 2
4	rs7533315	11860683	C/T	C		Intron 3
5	rs4846052	11857951	С/Т	T		Intron 4
6	rs1801133	11856378	С/T	C	Non-synonymous (222 Val/Ala)	Exon 5
7	rs6541003	11855867	A/G	G		Intron 5
8	rs2066462	11854896	C/T	C	Synonymous (352 Ser/Ser)	Exon 7
9	rs1801131	11854476	A/C	A	Non-synonymous (429 Ala/Glu)	Exon 8
10	rs17375901	11852516	C/T	C		Intron 9
11	rs2274976	11850927	А/G	G	Non-synonymous (594 Gln/Arg)	Exon 12
12	rs1537516	11847861	С/Т	C		3’-untranslatable region

**Fig. 1:**
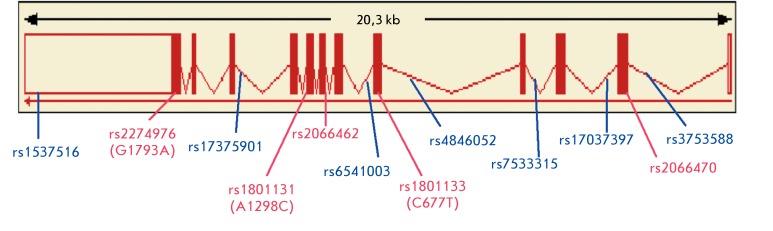
Localization of the SNPs studied in the *MTHFR * gene *. * The blue color indicates the polymorphisms located in introns and 3’UTR; red, in exons.

In this study, the methylenetetrahydrofolate reductase ( *MTHFR* ) gene was selected for use as a locus to investigate the LD structure in populations of various ethnic origins. According to the results of numerous studies, the polymorphic variants of this gene are associated with the development of a number of complex conditions, such as cardiovascular and oncological diseases, neural tube defects, abnormal pregnancy, as well as other pathological processes. The enzyme methylenetetrahydrofolate reductase catalyzes the only intracellular reaction of formation of 5-methyltetrahydrofolate, which is required for homocysteine (HC) conversion into methionine. A decrease in the activity of this enzyme is frequently caused by mutations in the *MTHFR* gene and results in the accumulation of HC and the development of moderate hyperhomocysteinemia.

A significant number of studies have been devoted to the role of genetic variability in the *MTHFR * locus. The results of these studies attest to the small contribution of individual SNPs of the *MTHFR * gene to the structure of inherited susceptibility to CD; moreover, the data for many ethnic groups are often contradictory. Since analysis of the LD structure in the candidate genes and identification of the haplotypes associated with the disease and their tagSNPs is considered to be one of the strategies for identifying the genetic variants underlying CD susceptibility with the highest potential [3, 16, 17], the assessment of the genetic variability of the candidate genes at the level of SNPs and haplotypes in different population samples is a rather topical task.

## Experimental

**Populations**

The data presented in this study were obtained via a molecular genetic DNA analysis of individuals from nine population groups inhabiting different regions of Northern Eurasia and belonging to seven ethnic groups ( *Table 1* ). The total sample included 837 individuals. The populations studied represent two Eurasian race types, Caucasian and Mongoloid, and speak languages of four linguistic families (Altaic, Paleo-Asiatic, Indo-European, and Uralic). Only individuals, nonmetisized in at least three generations, participated in the study. Ethnicity, genealogy, and membership of the individuals in sub-ethnic groups (in some cases) were ascertained on the basis of questionnaires. The sample of Tuvinians was collected in the Tuva Republic (settlement of Bai-Taiga). Two Buryat populations were examined in the Buryat Republic (the city of Ulan-Ude and settlement of Khuromsha). The Yakut sample was collected in the settlement of Dyupsya located in the eastern Ust-Aldan ulus (district) of Sakha Republic (Yakutia). The Central Asian populations are represented by Kyrgyz. Two Kyrgyz samples were made up of northern (the settlements of Kegety and Taldy-Su) and southern (the city of Osh) populations of the Republic of Kyrgyzstan; they belong to different sub-ethnic groups. The Russian sample was represented by residents of Tomsk. The Ket population was collected in the settlement of Kellog, Turukhanskii district, Krasnoyarsk krai. The Khanty population was collected in the settlement of Russkinskii, Khanty-Mansi Autonomous Okrug.

The analysis also included data on the Caucasian (residents of the state of Utah, USA), Chinese (residents of Beijing, China), Japanese (residents of Tokyo, Japan), and Yoruba (residents of Ibadan, Nigeria) populations presented in the HapMap database [11].

**Polymorphisms**

The following 12 SNPs of the *MTHFR* gene were selected for use as markers to study LD patterns: rs3753588, rs2066470, rs17037397, rs7533315, rs4846052, rs1801133 (C677T), rs6541003, rs2066462, rs1801131 (A1298C), rs17375901, rs2274976 (G1793A), and rs1537516 ( *Fig. 1* ). *Table 2* briefly characterizes the studied loci of the *MTHFR* gene. Ten of 12 SNPs resulted from the transitions (3 A→G and 7 C→T), two SNPs resulted from the transversions (A→С). The selected polymorphic variants are distributed in a relatively uniform manner over the gene sites (exons, introns, and 3’-untranslated regions); the minor allele frequency in most loci is at least 5% (according to the data from the NCBI database). Genotyping was carried out in accordance with the previously described protocols [18–20].

**Methods for the statistical processing of the results**

Statistical analysis was performed using conventional software packages: Statistica 6.0, ARLEQUIN, and Haploview 4.0. The distribution pattern of the resulting data was determined using the Kolmogorov–Smirnov test; haplotype frequencies were determined using the EM algorithm. The LD between SNP pairs was assessed using the Levontin’s D’ coefficient and Pearson’s correlation coefficient r ^2^ . The block structure was determined using the Solid Spine of the LD algorithm [21] provided by the Haploview 4.1 software, with the specified D’ threshold ≥ 0.8. The levels of genetic diversity and interpopulation differentiation were calculated via an analysis of the molecular variation (AMOVA). The selective neutrality of polymorphisms was studied using the Ewens–Watterson test [22]. The role of selection pressure in the formation of LD patterns and the level of genetic diversity in the populations was assessed using the conventional Tajima’s and Fu’s statistic tests of neutrality [23, 24].

## RESULTS and DISCUSSION

**Genetic diversity and haplotype structure at the **

*MTHFR* **locus in populations**

The gene pool of modern human populations was formed as a result of sequential evolutionary demographic processes: continuous evolution of genetic diversity in Africa and population variance as modern humans migrated, with partial isolation and reduction of the gene flow in inverse proportion to the migration distance. In different geographic areas, the populations have both a common and unique evolutionary history; the “fingerprints” of these histories can be observed in the modern human genome as LD patterns [3, 25–27].

The distribution of genotypes and the allele frequency, the observed heterozygosity, and the significance of goodness-of-fit of the *MTHFR* gene SNPs to Hardy–Weinberg proportions are presented in *Table 3* . All 12 loci appeared to be polymorphic in almost all the populations analyzed (with the exception of rs2066470 in the Ket population). The minor allele frequency varied from 0 to 39%; seven SNPs (rs3753588, rs7533315, rs4846052, rs1801133, rs6541003, rs1801131, and rs1537516) were identified in all the populations with a frequency higher than 5%. The resulting data lie within the range of variations of allele frequencies and genotypes of *MTHFR* polymorphisms which had been previously published and listed in the databases of the Caucasian and Mongoloid populations. In all the samples, the distribution of the genotype frequencies of almost all markers fitted into the Hardy–Weinberg proportions (with the exception of loci rs17375901, rs2066470, rs3753588, rs2274976, and rs1537516 in the Buryat subpopulation from the settlement of Khuromsha). Low and medium heterozygosity values were observed in the majority of cases, which was consistent with the world data. The highest heterozygosity for the loci combination was detected in the Yakut population (0.28); the lowest heterozygosity value was recorded in the residents of the settlement of Khuromsha (0.18). It is obvious that these values do not represent the SNP heterozygosity level of the populations examined, since the number of the loci taken into account was too small. These values are of some interest as they provide information pertaining to the degree of polymorphism in the *MTHFR* gene. As for the deviation from the Hardy–Weinberg proportions observed in this study, it could be a result of the shift in the estimated frequency values due to the small size of the sample. On the other hand, the cases of a reliable deviation of the distribution from the expected one may represent the specificity of population-genetic processes in the population, which can be associated both with the parameters of the genetic-demographic structure of the population and with the linkage with a functionally significant locus. We consider the latter reason to be more plausible.

The С677Т (rs1801133) polymorphic variant is one of 12 SNPs in the *MTHFR* gene which has been best studied. The missense mutation in С677Т (substitution of cytosine by thymine at position 677) results in alanine replacement for valine in the enzyme catalytic domain. In individuals homozygous and heterozygous for the polymorphic allele, the *in vitro* activity of the enzyme is reduced by 70% and 35%, respectively. The 677Т mutant allele frequency in world populations varies from total absence in the Dendi tribe to 55% in Spanish populations [28–30]. In Russia, the frequency of the 677Т allele is 29% in residents of the Moscow region and 32% in residents of Siberia [31, 32]. In the examined populations, the frequency of this allele varies from 12% in the Ket population to 31% in the Russian sample.

**Table 3:** Distribution of genotypes and minor alleles of the polymorphic variants of the *MTHFR * gene in the samples under study

№	SNPs studied	Genotype,allele	Frequency, %								
			Tuvinians(*N*=134)	Southern Kyrgyzes(*N*=111)	Northern Kyrgyzes(*N*=85)	Kets(*N*=38)	Buryats,city of Ulan-Ude (*N*=60)	Buryats, settlement of Khuromsha (*N*=60)	Yakuts (*N*=81)	Khanty (*N*=142)	Russians (*N*=126)
1	2	3	4	5	6	7	8	9	10	11	12
1	rs3753588	AA	2	1	1	0	0	3	4	2	1
		AG	13	14	22	11	17	5	20	28	18
		GG	85	85	77	89	83	92	76	70	81
		A	8	8	12	5	8	6	14	16	10
		*H*_e_	0.16	0.15	0.23	0.10	0.17	0.13	0.24	0.28	0.18
		p	0.21	0.48	1.00	1.00	1.00	0.008	0.15	1.00	1.00
2	rs2066470	CC	90	85	78	100	83	92	76	79	83
		CT	10	14	18	0	17	5	19	19	16
		TT	0	1	1	0	0	3	5	2	1
		T	5	8	10	0	8	6	14	11	9
		*H*_e_	0.13	0.16	0.20	0	0.17	0.13	0.25	0.20	0.16
		p	1.00	0.53	1.00	0	1.00	0.007	0.05	0.67	1.00
3	rs17037397	АА	0	0	0	0	0	0	3	0	0
		AC	12	11	8	16	20	5	17	26	11
		CC	88	89	92	84	80	95	80	74	89
		A	6	5	4	8	10	3	11	13	6
		*H*_e_	0.12	0.11	0.09	0.17	0.20	0.07	0.20	0.23	0.11
		p	1.00	1.00	1.00	1.00	1.00	1.00	0.24	0.13	1.00
4	rs7533315	CC	55	69	60	68	78	63	63	75	53
		CT	38	29	39	29	19	35	37	23	42
		TT	7	2	1	3	3	2	0	2	5
		T	26	16	21	17	13	19	18	13	26
		*H*_e_	0.38	0.28	0.34	0.31	0.24	0.32	0.31	0.23	0.38
		p	1.00	0.74	0.18	1.00	0.21	0.67	0.06	0.72	0.36
5	rs4846052	CC	46	55	40	55	58	58	40	51	30
		CT	41	42	52	39	36	35	53	39	53
		TT	13	3	8	6	6	7	7	10	17
		T	34	24	34	25	23	24	34	29	43
		*H*_e_	0.45	0.37	0.46	0.38	0.35	0.38	0.45	0.41	0.50
		p	0.33	0.12	0.23	1.00	1.00	0.73	0.13	0.68	0.47
6	rs1801133(C677T)	CC	67	53	53	79	72	55	61	67	50
		CT	28	37	44	18	25	42	33	29	37
		TT	5	10	3	3	3	3	6	4	13
		T	19	28	24	12	16	24	23	18	31
		*H*_e_	0.32	0.41	0.38	0.24	0.27	0.37	0.36	0.29	0.44
		p	0.26	0.36	0.14	0.41	0.62	0.48	0.75	0.57	0.10
7	rs6541003	AA	43	54	38	53	60	58	37	49	29
		AG	46	43	52	42	35	35	52	40	56
		GG	11	3	10	5	5	7	11	11	15
		G	34	24	36	26	23	24	32	31	43
		*H*_e_	0.45	0.38	0.47	0.39	0.35	0.38	0.47	0.43	0.50
		p	0.85	0.12	0.34	1.00	1.00	0.73	0.47	0.44	0.28
Table 3 (Contd.)											
1	2	3		5	6	7	8	9	10	11	12
8	rs2066462	CC	94	88	93	89	97	97	89	74	94
		CT	6	12	7	11	3	3	11	26	6
		T	3	6	3	5	2	2	6	13	3
		*H*_e_	0.06	0.12	0.07	0.10	0.05	0.05	0.11	0.23	0.06
		p	1.00	1.00	1.00	1.00	1.00	1.00	1.00	0.13	1.00
9	rs1801131(A1298C)	AA	44	62	34	58	60	58	46	51	40
		AC	35	35	54	37	36	35	48	38	48
		CC	21	3	12	5	4	7	6	11	12
		C	38	20	39	24	21	24	30	30	36
		*H*_e_	0.38	0.33	0.49	0.37	0.33	0.38	0.43	0.42	0.47
		p	0.79	0.56	0.25	1.00	1.00	0.73	0.29	0.32	0.69
10	rs17375901	CC	98	96	89	97	98	94	94	94	91
		CT	2	4	11	3	2	3	6	6	9
		TT	0	0	0	0	0	3	0	0	0
		T	1	2	5	1	1	5	3	3	4
		*H*_e_	0.02	0.04	0.11	0.03	0.03	0.11	0.07	0.06	0.09
		p	1.00	1.00	1.00	1.00	1.00	0.003	1.00	1.00	1.00
11	rs2274976(G1793A)	AA	0	0	0	0	0	3	3	1	0
		AG	10	30	11	8	17	4	17	23	13
		GG	90	70	89	92	83	93	80	76	87
		A	5	15	5	4	8	5	13	13	6
		*H*_e_	0.11	0.26	0.11	0.10	0.17	0.11	0.20	0.23	0.12
		p	1.00	0.12	1.00	1.00	1.00	0.003	0.24	1.00	1.00
12	rs1537516	CC	84	85	80	89	83	92	80	69	82
		CT	16	14	20	11	17	5	17	29	17
		TT	0	1	0	0	0	3	3	2	1
		T	8	8	10	5	8	6	13	16	10
		*H*_e_	0.15	0.16	0.18	0.13	0.17	0.013	0.20	0.27	0.18
		p	1.00	0.53	1.00	1.00	1.00	0.007	0.24	0.53	0.34
Average*H*_o _across 12 loci			0.21	0.24	0.27	0.19	0.21	0.18	0.28	0.27	0.27
Average*H*_e _across 12 loci			0.23	0.23	0.26	0.19	0.21	0.20	0.27	0.27	0.23

Note: *N* – the number of individuals per sample; *H*_e_ – expected heterozygosity; *Н*_о _ – observed heterozygosity; *p* – significance of goodness-of-fit to Hardy–Weinberg proportions. The statistically significant differences are indicated in bold.

The А1298С transition (rs1801131) resulting in the replacement of a glutamic acid residue by alanine in the enzyme regulatory domain is the second common polymorphism in the *MTHFR* gene. The enzymatic activity is reduced in individuals carrying the 1298С allele, although this reduction is not as significant as that in the ones carrying the 677T allele. According to some studies, MTHFR activity falls by 40–50% and a biochemical profile similar to that in homozygous carriers of the 677T allele is observed in compound heterozygous individuals [33]. The lowest frequency of the 1298C allele was detected in residents of Senegal (4%), whereas the highest frequency was detected in the Israeli and New Guinean populations (41%) [34, 35]. In Russians, the frequency of this allele varies from 24 to 38% [31]. In the populations under study, allele C occurs with a frequency ranging from 20% (in the southern Kyrgyzes) to 39% (in the northern Kyrgyzes).

**Fig. 2:**
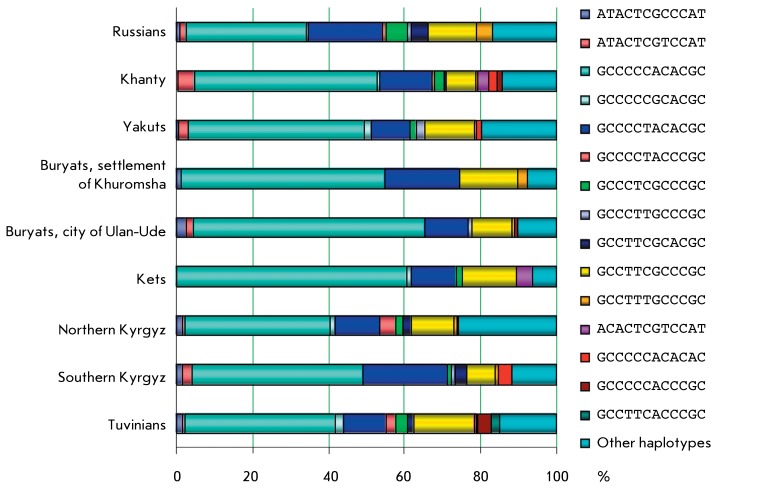
The distribution of haplotypes in the populations studied.

It has recently been ascertained that another *MTHFR * gene SNP, rs2274976 (G1793А), impacts the HC level. This locus has been subjected to less investigation in comparison with С677Т and А1298С. It is a known fact that the frequency of missense mutation G1793А varies from 1.3% in the Ashkenazi Jewish population to 26.6% in the Indonesian populations of Java [36]. It has been demonstrated that G1793А homozygosity results in an increase in the blood HC level by 40% [37, 38]. In the populations under analysis, the minimum frequency of allele A was observed in the Ket population sample, whereas the maximum frequency of the allele (15%) was observed in the southern Kyrgyz population.

A total of 160 haplotypes were detected in our samples; the theoretically possible number was 4,096. The maximum number of haplotypes was revealed in the Tuvinian population (47); the minimum number was revealed in the Ket population (11). A high level of haplotype diversity was observed in most samples, bar the Ket, Buryat, and southern Kyrgyz populations. If each mutation that resulted in the formation of a fixed polymorphic variant is assumed to be a unique event, and the mutation rate is assumed to be negligibly low, it should be postulated that only 12 haplotypes could be formed as a result of the mutation process. In this case, a considerable part of the haplotype diversity even in such a physically small genome region as an approximately 20-thousand-bp long *MTHFR * locus should have been formed due to the recombination events (see below).

The distribution of haplotypes occurring in the populations studied with a frequency of over 2% is presented in *Fig. 2* . The haplotypes with a frequency of more than 5% are referred to as the major ones. Three major haplotypes, GCCCCCACACGC, GCCCCTACACGC, and GCCTTCGCCCGC, were detected in all the populations studied, bar the Russian sample; the sum of their frequencies is more than 83% of the chromosomes observed in the Ket and Buryat populations, and more than 61% in the other samples. The degrees of haplotype diversity detected in the populations studied were different; nevertheless, all the samples contained identical major haplotypes, attesting to the fact that there can indeed be a common mechanism for the formation of the LD patterns.

**Architecture of linkage disequilibrium for the **

**Fig. 3:**
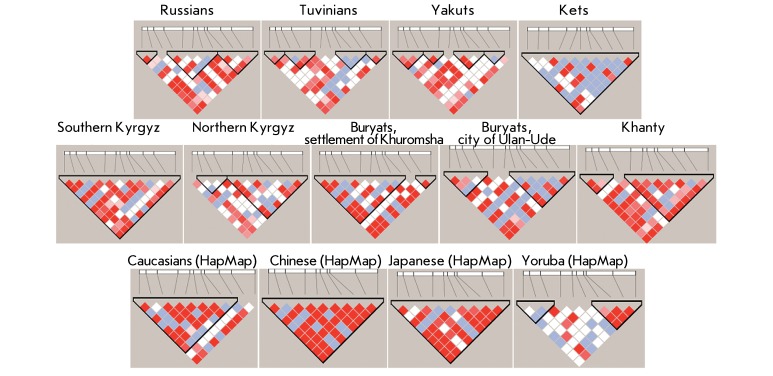
The structure of linkage disequilibrium in the *MTHFR* gene in the populations studied. The color scheme shows the strength of adhesion between SNPs: bright red – a strong link (D’=1, LOD>2), red and pink – a significant link (D’<1, LOD>2), white – poor link (D’<1, LOD<2). The lilac cell denotes the impossibility to calculate the linkage disequilibrium due to the low frequency of the minor allele polymorphism (D’=1, LOD<2). Localization of SNPs for each population is as follows (from left to right): 1 – rs3753588, 2 – rs2066470, 3 – rs17037397, 4 – rs7533315, 5 – rs4846052, 6 – rs1801133, 7 – rs6541003, 8 – rs2066462, 9 – rs1801131, 10 – rs17375901, 11 – rs2274976, 12 – rs1537516 (there is no polymorphism rs3753588 in the populations from the HapMap project). In the Yoruba population, marker rs17375901 was excluded from the analysis because of its monomorphicity.

*MTHFR* **gene in certain Eurasian populations**

The structure of LD between the investigated loci of the * MTHFR* gene in 13 population samples is shown in *Fig. 3.* The maximum linkage between the SNPs studied was demonstrated for the southern Kyrgyz, Ket, Chinese, and Japanese populations. In these samples, all the allelic variants of the *MTHFR* gene under analysis belong to the same haplotype block. A single block was also observed in the Caucasian individuals from the HapMap project; however, it did not contain the rs2274976 and rs1537516 markers. Two blocks were detected in the northern Kyrgyz population: the first one comprising three SNPs (rs2066470, rs17037397, and rs7533315), the second one encompassing a 10-thousand-bp-long region and containing eight SNPs. Two blocks were also revealed in the Khanty population: the first one containing rs3753588, rs2066470, and rs17037397; the second one, identical to block № 2 in the northern Kyrgyz population. Strong linkage between the first nine SNPs belonging to the first block (9-thousand-bp long), as well as that between rs2274976 and rs1537516 forming the second small block were revealed in the Buryat population of the settlement of Khuromsha. A significant linkage between many polymorphic variants was also detected in the Buryat population of the city of Ulan-Ude; however, two different blocks (3- and 8-thousand-bp long) were represented in the LD structure of this population. Three blocks were observed for the Russian population: the first block was made up of two closely located SNPs (rs3753588 and rs2066470), the second block comprised five polymorphisms (rs7533315, rs4846052, rs1801133, rs6541003, and rs2066462), whereas the third block was made up of four SNPs (rs1801131, rs17375901, rs2274976, and rs1537516).

Four small haplotype blocks consisting of two or three neighbouring SNPs can be detected in the Tuvinian and Yakut populations. Two blocks were observed in the Yoruba populations: the first one consisted of 2 thousand bp and contained rs2066470, rs17037397, and rs7533315; the second block was appreciably long (7 thousand bp) and comprised four loci (rs2066462, rs1801131, rs2274976, and rs1537516). Thus, the haplotype blocks (with lengths varying from 847 bp to 16 thousand bp) were represented by several (3–6) major haplotypes, the sum of which provided more than 90% of the chromosomes observed. The composition and size of the blocks varied depending on the population structure. It is particularly remarkable that the functionally significant С677Т and А1298С polymorphisms were not linked in all the populations, bar the southern Kyrgyz and Ket samples, as well as populations from the HapMap project. It should also be noted that close linkage between the SNPs that form the second block in the Yoruba population was observed in many populations.

At the time of writing, the degree of haplotype diversity and length of linkage blocks in various genome regions have been identified in many human populations [39, 40]. A considerable variability of the haplotype structure was revealed; it was considerably different when passing from one genome region to another and frequently alternated with the regions with a low LD level. The length of haplotype blocks varied from 1 to 100 (and more) thousand bp [9]. Some researchers have reported on the consistent spatial distribution of the haplotype blocks in several human genome regions, referring to the common mechanism of formation of these blocks in various populations as the possible reason for this phenomenon [3, 41]. An African versus non-African dichotomy was also described in several segments of the human genome [42, 43], the haplotype blocks with the greatest length in non-African populations (> 44 thousand bp) in comparison with those in African populations (> 22 thousand bp) [9]. Patterns of meiotic recombination were revealed, attesting to the fact that the haplotype blocks are confined by the regions with a low LD level corresponding to those with high recombination indices [10, 44]. The relationship between the recombination frequency, chromatin structure, and the various structural and functional components of the nucleus were also assessed within the framework of intense research relating to the recombination in the human genome. It was demonstrated via this analysis that various potential factors, both local (DNA nucleotide sequence, chromatin structure) and the ones unrelated to the structure of the recombination site directly, may affect the recombination indices of a specific chromosome segment. An assumption was made that recombination is the major causal factor accounting for the formation of the linkage blocks resulting from the disintegration of long haplotypes in chromosome regions with a comparatively high recombination level. This hypothesis was confirmed by the fact that several genome regions in various populations possess an identical LD structure [45–47]. Meanwhile, data have been obtained attesting to a significant interpopulation variation in the degree and pattern of LD within the same genome region [10, 48–50]. These results attest to the fact that the pattern of LD revealed for a specific population or sample presumably cannot be automatically extrapolated onto other populations, at least in particular genome regions. It is unlikely that one common map of linkage disequilibrium in the genome will appear to be useful for the selection of genetic markers for performing association studies in a number of populations, since the interaction between various population-specific factors and genome-specific mechanisms upon the formation of the LD structure cannot be neglected.

The population-specific nature of the formation of LD patterns was confirmed in this study. The strongest linkage (all SNPs belong to the same block) between the loci under study was detected in the Ket, southern Kyrgyz, Chinese, and Japanese populations. Close linkage was also observed in the Buryat populations. Two explanations to the observed retention of block structure and length can be proposed: either common ancestors or positive selection; the latter frequently resulting in an increase in the length of the block containing a useful allele [51]. Since the minimum number of haplotypes among all the populations investigated was revealed in the Ket and Buryat samples, there is a probability that the ancestral effect could have taken place in this specific case. However, a number of other factors also affect the increase in the LD structure [49]; namely, genetic isolation, population subdivision or mixing, balancing selection, the bottleneck effect, small population size, and other reasons. The influence of the aforementioned factors on certain populations cannot be ignored.

It has been demonstrated that the length of LD patterns in the human genome determines the potential and design of association studies that use SNPs for the mapping of the genes underlying complex indices. According to the current estimations, the number of markers required for a LD-based genome scan of different populations varies from 120, 000 to several millions and is attestable to the following facts: the cost of genotyping is tremendous, and there can be problems with the validity of the statistical conclusion. An assumption was made that the number of markers required for CD mapping will be considerably lower in populations with a high degree of LD [52].

**Selection of tagSNPs in the **

*MTHFR* **gene**

According to the estimations made by different researchers, the human genome contains more than 7.5 million common SNPs with a minor allele frequency (MAF) of at least 5% [21, 53], which are partly responsible for the inherited risk of developing many CDs. Today, tagSNP selection aimed at broadening the genetic coverage is one of the most efficient strategies for designing a genetic marker panel for analyzing the association with CD [54]. In this case, the gene coverage is defined as the percentage of the set of all common SNPs with MAF of 5% that demonstrate threshold correlation with at least one SNP from the specified polymorphism array [55]. The tagSNP approach has advantages due to knowledge of the LD block structure of the human genome. Moreover, this strategy considerably reduces effort and cost in genotyping [53, 56]. Because of the undeniable potential benefit of tagSNP selection for association studies, a proposition was made that tagSNPs be efficiently identified using various algorithms.

**Fig. 4:**
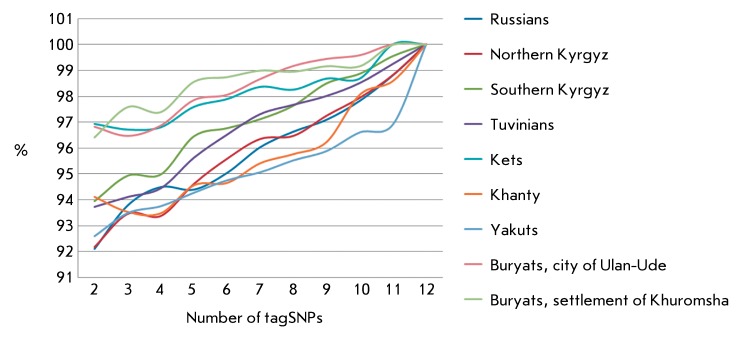
Variability in accuracy in predicting set tagSNPs for the *MTHFR* gene, depending on their number and population sample *.*

Two methods (namely, STAMPA and Tagger) were used in this study for tagSNP identification. The genotype data are used in the STAMPA method, whereas no data on the haplotypes and block architecture of the genome region under study are required [57]. This algorithm is based on the hypothesis that the correlation between SNPs tends to decrease as the physical distance between them increases; the allelic SNP variant can be determined based on knowledge of the allelic variants of the nearest tagSNPs from each side. A prognostic significance of more than 90% is achieved in all populations already when two tagSNPs of the *MTHFR* gene are selected ( *Fig. 4* ). This is presumably because the gene is small and the polymorphic sites are located physically close to each other. However, to achieve a prognostic significance of 99–100%, the number of tagSNPs needs to be considerably high. In our opinion, this fact is a significant drawback of this algorithm.

The tagSNPs in the *MTHFR* gene were also identified in this study using the Tagger algorithm provided by the HaploView software [21]. An aggressive tagging algorithm based on tagSNP identification in two- and three-marker haplotypes, within which all the polymorphic variants should be closely linked with one another (LOD > 3), was used in this method [26]. The tagSNPs detected using the aforementioned algorithm are listed in *Table 4.*

The prognostic significance of this set of tags with respect to the entire polymorphism array under study is equal to 100%. According to the results obtained, unequal tagSNP sets were observed in different populations at the specified threshold of prognostic significance, which was presumably caused by the variation of the LD structure and the haplotype diversity of the * MTHFR* gene within the samples. This fact was confirmed by the statistically significant correlation between the haplotype diversity and the variability of the number of tagSNPs (r ^2^  = 0.85; *p * < 0.01). The problem of the possible transfer of tagSNPs from one population to another is rather significant due to the considerable topicality of association analysis using tagSNPs identified on the basis of the HapMap project data. An appreciably high prognostic significance of tagSNP sets in several genome regions selected for CEU, CHB, and JPT with regard to the Caucasian and Mongoloid populations has been recently shown in a series of experiments [58–60]. Nevertheless, it has been determined that the extrapolation level of tagSNP decreases when the set of tagSNPs established for CEU populations is used in association studies in the African and several isolated Caucasian populations [26, 61, 62]. At the same time, it has been demonstrated that the most universal tagSNPs providing maximum genetic coverage in the other populations are found in the Yoruba population because of the minimum strength of LD in this sample [53].

**Table 4:** *MTHFR* gene tagSNPs identified using the Tagger algorithm

Population	Number of tagSNPs	*MTHFR *gene SNPs											
Russians	9	1	2	3	4	5	6	7	8	9	10	11	12
Northern Kyrgyz	11	1	2	3	4	5	6	7	8	9	10	11	12
Southern Kyrgyz	8	1	2	3	4	5	6	7	8	9	10	11	12
Tuvinians	11	1	2	3	4	5	6	7	8	9	10	11	12
Kets	8	1	2	3	4	5	6	7	8	9	10	11	12
Khanty	9	1	2	3	4	5	6	7	8	9	10	11	12
Yakuts	10	1	2	3	4	5	6	7	8	9	10	11	12
Buryats, city of Ulan-Ude	8	1	2	3	4	5	6	7	8	9	10	11	12
Buryats, settlement of Khuromsha	7	1	2	3	4	5	6	7	8	9	10	11	12

Note: The following SNP numeration is used: 1 – rs3753588, 2 – rs2066470, 3 – rs17037397,

4 – rs7533315, 5 – rs4846052, 6 – rs1801133, 7 – rs6541003, 8 – rs2066462, 9 – rs1801131, 10 – rs17375901,

11 – rs2274976, 12 – rs1537516. The *MTHFR* gene tag SNPs are shown in bold type at grey background.

**Table 5:** Comparative characteristics of STAMPA and Aggressive tagging algorithms for tagSNP determination

Population	90% prognosis accuracy		95% prognosis accuracy		98% prognosis accuracy		100% prognosis accuracy		Haplotype diversity	Number of blocks
	STAMPA	Tagger	STAMPA	Tagger	STAMPA	Tagger	STAMPA	Tagger		
	Number of*MTHFR*gene tagSNPs									
Russians	2	8	6	9	12	9	10	9	0.69	3
Northern Kyrgyz	2	10	6	11	12	11	10	11	0.77	2
Southern Kyrgyz	2	7	4	8	12	8	9	8	0.62	1
Tuvinians	2	10	5	11	12	11	9	11	0.82	4
Kets	2	7	2	8	12	8	10	8	0.49	1
Khanty	2	8	7	9	12	9	10	9	0.78	3
Yakuts	2	9	7	10	12	10	12	10	0.72	4
Buryats, city of Ulan-Ude	2	7	2	8	12	8	5	8	0.59	3
Buryats, settlement of Khuromsha	2	6	2	7	12	7	5	7	0.55	2

A comparative analysis of the efficiency of STAMPA and Tagger algorithms depending on the level of prognostic significance was carried out in this study. It is clear from *Table 5* that the minimum number of tagSNPs at a prognostic significance of 90–95% is determined using the STAMPA algorithm, whereas the Tagger method is more efficient at a prognostic significance of 98–100%.

TagSNPs are widely used in various genetic studies as a tool that efficiently represents genetic diversity. Nevertheless, the quality of the selected tagSNPs depends on the original array in which they were characterized. If the original marker density was low, the selected tagSNP will “capture” less information than is required for the analysis. The required marker density in the initial data array varies within different genome regions depending on a number of factors, such as the recombination level, LD structure, SNP frequencies, mutation character, and the demographic history of a population [17].

**Phylogenetic analysis of the relationships between haplotypes at the**

**Fig. 5:**
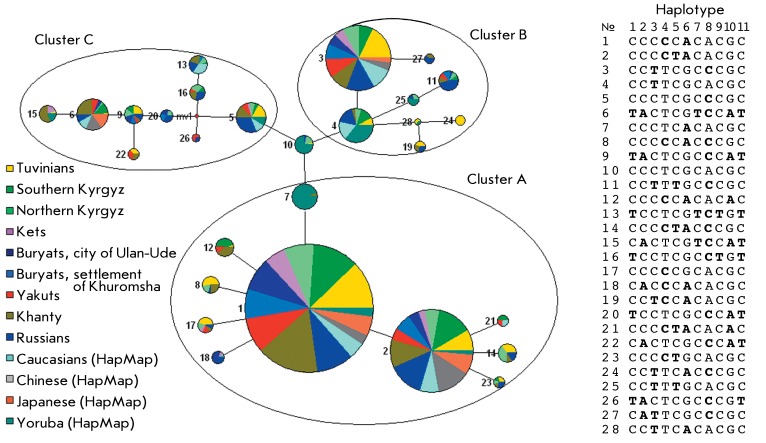
The median tree of the haplotypes occurring with a frequency of more than 0.1% in the total sample. Mutant alleles are shown in bold letters; ancestral haplotype is denoted as №10. Node diameter represents haplotype frequency in the total sample. Numeration of SNPs in haplotypes is as follows: 1 – rs2066470, 2 – rs17037397, 3 – rs7533315, 4 – rs4846052, 5 – rs1801133, 6 – rs6541003, 7 – rs2066462, 8 – rs1801131, 9 – rs17375901, 10 – rs2274976, 11 – rs1537516.

*MTHFR* **locus and the assessment of the selective neutrality of the polymorphisms under study**

The phylogenetic analysis of the relationships between the haplotypes, which are determined on the basis of diallelic markers and characterized by a frequency of occurrence in the total sample of more than 0.1%, were carried out by constructing phylogenetic trees (networks) of haplotypes using the median network algorithm implemented in the Network software. The haplotype consisting of ancestral alleles was used as the ancestral haplotype (the data was taken from the NCBI database). The results obtained attest to the fact that all the haplotypes observed in the human populations under analysis originated from a common ancestral variant, which occurs in the Yoruba population (with a frequency of approximately 12%) and in the Russian and southern Kyrgyz populations (with a frequency of 1%) (haplotype 10 in *Fig. 5* ). This fact supports the hypothesis of the Recent African Origin of modern humans. The fact that haplotypes № 4 and 7, which are the closest ones to the ancestral variant, occur in the Yoruba population with a significant frequency provides further potency to this hypothesis.

All the haplotypes observed lie within six mutation steps from their common ancestor and can be subdivided into three major clusters, A, B, and C, which are formed from the CC **T** TCGCACGC, CCCTCGC **C** CGC, and CCCTC **A** CACGC haplotypes (№ 4, 5, and 7, respectively; *Fig. 3* ). Cluster A is represented by 10 haplotypes, two of which (№ 1, 2) are the most widespread (their total frequency being more than 50% of the frequency of all haplotypes in the total sample) and were revealed in all the populations studied. It should be noted that the tree structure in this cluster is of a strongly pronounced star-shaped character, which obviously indicates a sudden population expansion in the demographic history. Cluster B contains eight haplotypes, including haplotype № 3, which is the third most frequently occurring haplotype and is represented in all the populations, bar the Yoruba sample. It is worthy of note that haplotypes № 8, 12, 17, 18, 21, 14, 23, 27, 11, 24, 19 of clusters A and B located at the branch tips of the phylogenetic tree occur only in northern Eurasian populations and presumably emerged relatively recently. Haplotypes belonging to cluster C contain a large number of mutant alleles; they are likely to have been formed as a result of recombination events.

Since the SNP mutation rate and their diversity observed in the modern populations can be assessed, it is possible to calculate the time of origin of this haplotype lineage. It has been known that diversity assessments based on the phylogeny of DNA nucleotide sequences are independent of demographic processes [63] and suitable for determining the evolutionary age of genetic lineages. The term “age” is understood as the coalescence time (coalescence to the common ancestor); i.e., as the generation time of the diversity observed. With allowance for these facts, the variation of the alleles of the same SNP in the same locus was considered to be a mutation step in order to assess the haplotype coalescence time. An identical mutation rate (1*10 ^–8^ mutations per locus per generation) was set for all the polymorphic variants [64]. The generation time was assumed to be equivalent to 20 years. The haplotype coalescence time was assessed using the Network software. In general, the age of diversity generation determined on the basis of 12 SNPs in the *MTHFR * gene was equal to 314,000±135,000 years. The median haplotype trees obtained by dividing the *MTHFR* gene into two blocks (the first one comprising rs3753588, rs2066470, rs17037397, rs7533315, rs4846052, rs1801133, rs6541003; and the second one comprising rs2066462, rs1801131, rs17375901, rs2274976, rs1537516) were analyzed in order to assess the accuracy of the results. This analysis was performed because of the fact that the specified regions of the *MTHFR* gene occur in different LD blocks in most populations, including the Yoruba sample. Therefore, an independent phylogenetic analysis of two blocks of the *MTHFR* gene may be less prone to the possible errors added because of recombination. Finally, the coalescence time for the first block turned out to be equal to 350,000±188,000 years; the age of the ancestral haplotype of the second block was assessed as 306,000±188,000 years.

Although the phylogenetic analysis that was performed is an appreciably powerful and efficient tool to characterize the evolutionary relationships between the haplotypes, it should be noted that particular care needs to be taken when interpreting the absolute assessments of the coalescence time (i.e., time expressed in years), since the key parameters underlying these assessments include the mutation rate and the absence of recombination within the genome region under analysis.

According to the results of a number of studies [65, 66], a relatively recent and rapid expansion of human populations from Africa left a considerable footprint on our genome by forming a structure of genetic variations in human populations, which is of biomedical significance, among other factors. It should be noted that the genomic variability causing the phenotypic difference between two individuals is only 0.1%. In fact, most of these variations in DNA should be evolutionary neutral; however, a great number of polymorphisms affecting the phenotype have been revealed, which can serve as selection objects or can be subjected to subsequent selection [65].

When analyzing the selective neutrality of *MTHFR * gene polymorphisms using the Ewens–Watterson test, neutrality deviation was detected only at rs4846052 and rs6541003 in the Russian samples (the F criterion observed for the Ewens–Watterson test was equal to 0.99 ( *р*  = 0.039) and 0.84 ( *р*  = 0.041), respectively) and the Caucasian populations from the HapMap project ( *F*  = 0.79 ( *р*  = 0.021) and *F*  = 0.98 ( *р * = 0.030), respectively). All three functionally significant SNPs causing an increase in the HC blood level turned out to be selectively neutral. This can presumably be attributed to the fact that even a certain variation in phenotype can be selectively neutral, provided that it does not affect reproductive efficiency [67]. Nevertheless, data concerning the selection of the 677T allele in the Spanish population have been reported. They were based on the study of the variation of the distribution of the frequencies of genotypes of alleles of the С677Т polymorphism during the XX century. An increase in the number of individuals with the 677TT genotype has been noted in the population. This increase was caused by the fact that many women took folic acid during the second and third trimesters of pregnancy, resulting in an increase in the viability of the carriers of the 677T allele during early stages of embryogenesis [68]. Furthermore, the selective significance of the T allele was supported by the results of the analysis of the distribution of the frequencies of alleles, genotypes, and haplotypes of the С677Т and А1298С polymorphisms in the *MTHFR * gene in the Israeli, Japanese, and African populations. According to these data, the 677T allele is found in the haplotypes with a selective advantage [69].

It is a known fact that the 677T allele frequency in world populations is very heterogeneous, varying from complete absence in the representatives of African tribes to 55% in Spanish populations [28–30]. Moreover, the allele frequency gradient is observed in Europe in the north–southward direction [70]. It has been demonstrated that the frequency of 677TT homozygous individuals in North America increases in the direction from Western Canada (Alberta) to the Southeastern United States (Atlanta), reaching a peak in Mexico [71]. The mechanisms of gradient generation have not as yet been reliably ascertained; however, there are at least three hypotheses, which are based on the assumption that the high 677T allele frequency is caused by the action of natural selection. The first hypothesis postulates that a decrease in MTHFR activity during the famine reduces homocysteine remethylation, thus saving monocarbon radicals in the tetrahydrofolate metabolism for essential DNA and RNA synthesis. According to the second hypothesis, the carriers of the mutant gene are less likely to develop colon cancer; therefore, the mutation frequency in the population may gradually increase [18]. The third hypothesis considers the gene–medium interactions between MTHFR and the folic acid content as the major factor for the accumulation of 677TT homozygous individuals in the population. Evidence supporting the latter theory was obtained in a number of experimental studies [34, 70, 71].

**Fig. 6:**
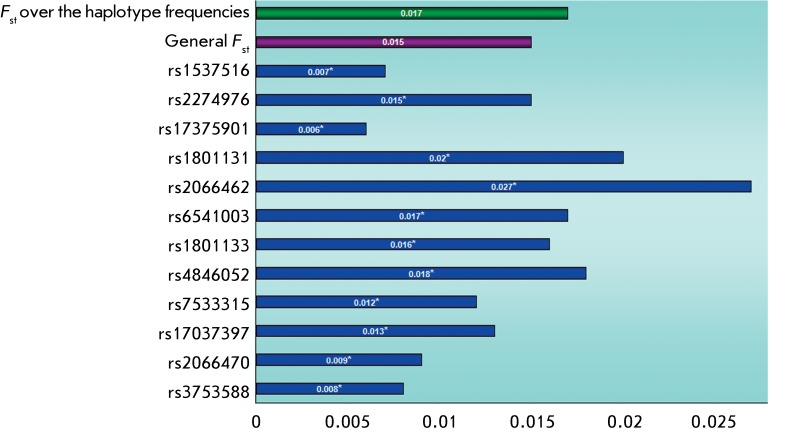
The overall genetic differentiation of the total sample studied over the polymorphisms of the *MTHFR* gene. Note: * indicates a statistically significant difference (р<0.05).

The role of selection pressure in the formation of LD patterns and the level of genetic diversity in populations was assessed using the standard statistical tests of neutrality proposed by Tajima and Fu [23, 24]. The value of Tajima’s D test turned out to be negative in all the populations studied; however, it was statistically insignificant. The value of the Fu’s *F* s test was negative and statistically significant in the populations of Tuvinians ( *F* s = –11.28, *р*  < 0.01), northern Kyrgyz ( *F* s = –24.15; *р* < 0.00001), Yakuts ( *F* s = –19.76, *р*  < 0.00001), and Khanty ( *F* s = –10.31, *р*  < 0.01), attesting to either a possible effect of negative selection on a specific genome region in these populations or population expansion. However, the mixing of these populations with neighboring ones could have also resulted in an increase in DNA diversity and the *F* s test.

In general, the data obtained attest to the fact that stabilizing selection has an impact on the rs4846052 and rs6541003 loci in the Caucasian populations from the HapMap project and that the negative selection possibly affects specific haplotypes of the *MTHFR* gene in the populations of Tuvinians, northern Kyrgyz, Yakuts, and Khanty. It should be noted that these four populations are characterized by a higher level of haplotype diversity (more than 70%) and low LD level among all the groups under analysis.

**Genetic differentiation and relationships between the populations under study**

The data relating to the degree of gene differentiation ( *F*_st_ ) in the total sample with respect to each of the markers selected are presented in *Fig. 6* . It should be noted that all the polymorphic variants studied demonstrated a reliable differentiation. It has been shown that the differences in allele frequencies at the rs4846052, rs1801133, rs6541003, rs2066462, rs1801131, and rs2274976 loci contribute most to interpopulation diversity. The lowest degree of interpopulation diversity is typical of rs17375901. The level of genetic differentiation of the populations under study with respect to the allele frequencies of the 12 *MTHFR* gene SNPs under investigation was equal to 0.015, and equal to 0.017 with respect to haplotype frequencies. The assessment was carried out using the *F*_st_ coefficient.

**Fig. 7:**
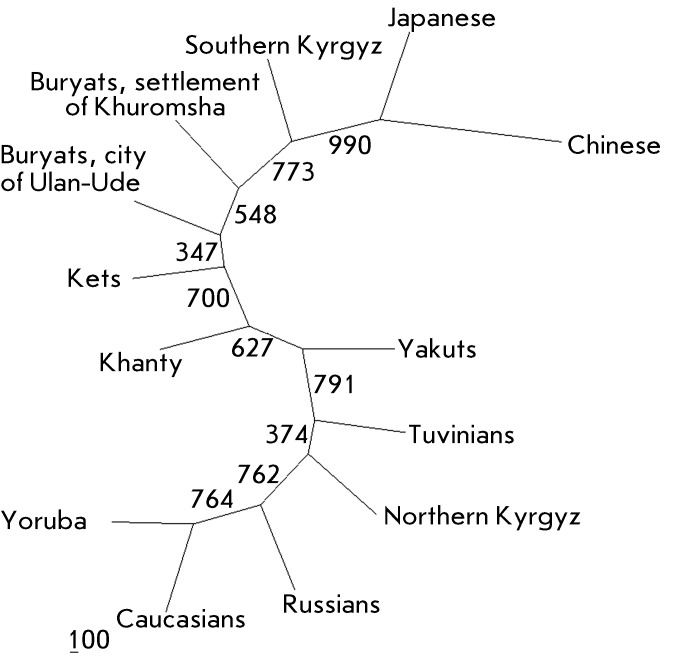
Dendrogram of the genetic relationships between the populations studied. The length of the branches of the phylogenetic tree corresponds to genetic distances.

The phylogenetic analysis of the genetic relationship between the populations was carried out based on a data array for 13 populations. The resulting dendrogram showing the genetic relationships between the populations is presented in *Fig. 7* . Two major clusters can be isolated in the phylogenetic tree. The first cluster comprises the Yoruba, HapMap Caucasians, northern Kyrgyzes, Tuvinians, and Yakuts; the second cluster consists of the Khanty, Ket, Buryat, southern Kyrgyz, Japanese, and Chinese populations. This approach reveals the considerable differences between the gene pools of the Caucasian (Russians, HapMap Caucasians) and Mongoloid (Japanese, Chinese, Buryats, southern Kyrgyzes) populations, as well as the close genetic relationship between the Yoruba and HapMap Caucasian populations, and the Chinese and Japanese populations. It is of interest that the northern and southern Kyrgyz are located in different clusters at a significant genetic distance. The resolution capacity of the tree constructed based on only 11 loci is insufficient to make any definite conclusions relating to the details of the genetic relationships between the populations under study and only attests to the degree of genetic variations between them with respect to the * MTHFR* locus.

## CONCLUSIONS

This investigation of the architecture of linkage disequilibrium of the *MTHFR* locus in nine populations inhabiting northern Eurasia was based on a conception assuming that the human genome has a block structure. The data relating to the Caucasian, Chinese, Japanese, and Yoruba populations obtained in the HapMap project were used as a basis for population comparisons.

A population-specificity of the LD structure of the *MTHFR* gene in various ethnoterritorial groups inhabiting northern Eurasia was revealed in this study. In addition, similarity in LD architecture was detected for certain populations, attesting to the role of evolutionary history in the organization of the block structure of LD.

Different degrees of haplotype diversity were established for the populations under study; nevertheless, identical major haplotypes were identified in all the samples with the exception of the Yoruba population, attesting to the fact that there may have been a common mechanism of formation of LD patterns in the *MTHFR* gene. The phylogenetic analysis of haplotypes showed that all the haplotypes observed in the populations under study originated from a common ancestral variant, thus attesting to the significant role of recombination in the generation of genetic diversity in the * MTHFR* locus and the possibility of a sudden population expansion. The age of generation of the diversity with respect to 12 SNPs of the *MTHFR * gene was 314,000±135,000 years.

Data supporting the impact of stabilizing selection on the rs4846052 and rs6541003 loci in HapMap Caucasian populations and that of negative selection on certain haplotypes of the *MTHFR* gene in populations of Tuvinians, northern Kyrgyz, Yakuts, and Khanty, which are characterized by the highest level of haplotype diversity (over 70%) and a low level of LD among all the groups under study, were also obtained in this work. All the investigated SNPs of the *МTHFR* gene showed reliable differentiation. Data on separate loci demonstrated that the variations with respect to allele frequencies at loci rs4846052, rs1801133, rs6541003, rs2066462, rs1801131, and rs2274976 contribute most to the interpopulation diversity. The lowest degree of interpopulation diversity is typical of the rs17375901 marker, which is monomorphic in the Yoruba population.

Thus, the architecture of LD in the human genome, and in the *MTHFR* locus in particular, is of a population-specific character and is to a significant extent determined by the evolutionary history of the population. It is obvious that the ethno-specific variability of haplotype blocks should be taken into consideration when analyzing the structure of LD and selecting tagSNPs during genetic mapping of common diseases both at the whole-genome level and for association studies, when a disease-associated variant is detected on the basis of its linkage with the nearest sites in a relatively narrow genome region. Subsequent investigation of the character of the genetic diversity and linkage disequilibrium in the genome of specific geographical, ethnic, or population groups will enable to reconstruct the genetic history of populations and detect the footprints of the natural selection associated with the adaptive variability. 

## Abbreviations

CD: complex diseases
LD: linkage disequilibrium
MTHFR: methylenetetrahydrofolate reductase
SNP: single nucleotide polymorphism
HC: homocysteine
